# Object-Based Image Analysis Applied to Low Altitude Aerial Imagery for Potato Plant Trait Retrieval and Pathogen Detection

**DOI:** 10.3390/s19245477

**Published:** 2019-12-12

**Authors:** Jasper Siebring, João Valente, Marston Heracles Domingues Franceschini, Jan Kamp, Lammert Kooistra

**Affiliations:** 1 Aurea Imaging, 3527 KT Utrecht, The Netherlands; 2 Laboratory of Geo-Information Science and Remote Sensing, Wageningen University & Research, 6708 PB Wageningen, The Netherlands; marston.franceschini@wur.nl (M.H.D.F.); lammert.kooistra@wur.nl (L.K.); 3 Information Technology Group, Wageningen University & Research, 6706 KN Wageningen, The Netherlands; 4 Wageningen Plant Research, Wageningen University & Research, 6708 PB Wageningen, The Netherlands; jan.kamp@wur.nl

**Keywords:** OBIA, VHR, erwinia bacteria, PVY virus, disease detection, color spaces

## Abstract

There is a growing demand in both food quality and quantity, but as of now, one-third of all food produced for human consumption is lost due to pests and other pathogens accounting for roughly 40% of pre-harvest loss in potatoes. Pathogens in potato plants, like the Erwinia bacteria and the PVY^NTN^ virus for example, exhibit symptoms of varying severity that are not easily captured by pixel-based classes (as these ignore shape, texture, and context in general). The aim of this research is to develop an object-based image analysis (OBIA) method for trait retrieval of individual potato plants that maximizes information output from Unmanned Aerial Vehicle (UAV) RGB very high resolution (VHR) imagery and its derivatives, to be used for disease detection of the *Solanum tuberosum*. The approach proposed can be split in two steps: (1) object-based mapping of potato plants using an optimized implementation of large scale mean-shift segmentation (LSMSS), and (2) classification of disease using a random forest (RF) model for a set of morphological traits computed from their associative objects. The approach was proven viable as the associative RF model detected presence of Erwinia and PVY pathogens with a maximum F1 score of 0.75 and an average Matthews Correlation Coefficient (MCC) score of 0.47. It also shows that low-altitude imagery acquired with a commercial UAV is a viable off-the-shelf tool for precision farming, and potato pathogen detection.

## 1. Introduction

The world’s population is expected to grow to almost 10 billion by 2050 [1], and a parallel income growth in low- and middle-income countries is hastening a dietary transition towards higher consumption of meat, fruits, and vegetables. The Food and Agriculture Organization (FAO) has projected that agriculture will have to produce almost 50 percent more food globally than it did in 2012 to meet this demand [2]. Similar leaps in agricultural productivity have occurred but have come at heavy costs to the natural environment with a severe loss of biodiversity, rapid depletion of natural resources, and an increase of global greenhouse gas emissions. These costs contribute to global warming, the spread of transboundary pests, deforestation, and desertification [2,3]. Subsequently, maintaining the pace of production increases via high-input, resource-intensive farming systems may be more difficult than in the past [2].

One-third of all food produced for human consumption is currently lost with pests and other pathogens accounting for roughly 40% of pre-harvest loss in potatoes [4]. Reducing this loss would grossly lessen the need for production increases, making early and accurate detection of these pathogens (and subsequent management) a key factor in securing global food security [4].

Currently there are several promising proximal sensor-based methods in early development that indirectly detect the spread of crop diseases, done by measuring either reflectance, temperature, or fluorescence in pixels associated to the respective plants [5]. Recent technical advancements in sensor sensitivity, material weight, computational capacity, and telemetry have also enabled very-high-resolution (VHR) remote sensing using unmanned aerial vehicles (UAV). This allows for more detailed and frequent data acquisition not previously possible with proximal sensing [6]. Recent review papers [7,8] present the added value of the VHR character of UAV-based imagery for precision agriculture. The suitability for applications related to the assessment of drought stress, weed detection, nutrient status, growth vigor, and yield prediction is rated as high.

Earlier studies have shown the use of UAV acquired RGB imagery for pathogen detection [9]. Plant pathogens however often exhibit symptoms of varying severity that are not easily captured by pixel-based classes alone because these ignore shape, color variability, and morphology in general. Soft rot pathogens for instance, such as the Erwinia bacteria, induce cell degradation which causes leaf discoloration, venial necrosis, black leg, growth stunting, and ultimately tuber degradation [10,11]. This means that the increase in within-class variance by VHR imagery actually decreases the potential accuracy of any purely pixel-based classification. This conflict is coined the H-resolution problem by [12]. Pixel-based classification itself has seen widespread use in agricultural research. Notable examples include: plant segmentation by means of Otsu thresholding on the excess green index (ExG), weed identification using support vector machines (SVM), late blight detection in tomatoes using spectral angle mapping, and the application of deep learning approaches for vine disease detection [5,13,14]. While good classification performance was achieved by all said methods, they all suffer under varying lighting conditions, shadows, and background complexity.

Object-based image analysis approaches came to the forefront in 2000 [15], which analyzed image-objects instead of individual pixels. These image-objects are pixels that are bundled by their varying levels of spectral, topological, or structural similarity [16,17]. UAV-based VHR imagery has reached a spatial resolution that effectively matches the objects of interest, i.e., sub-centimeter and context-sensitive plant traits. Arguments have been made that image analysis in these situations should forgo pixel-based approaches in favor of a more object-based approach that could potentially better capture these subtle classes [18,19,20,21].

This paper addresses the problem of approximating semantic object classes from a UAV-based VHR image by their morphological features to identify potato plant pathogens. The proposed approach is split into two parts; object-based mapping of potato plants using large scale mean-shift segmentation (LSMSS), and classification of disease in said objects using their morphological traits. The former explores the application of color spaces, following [22] who evaluated them on their ability to optimize segmentation of vegetation in crop site imagery using a naive Bayesian classifier and supervised sampling. The latter explores the viability of disease detection through characterizing approximated potato plants with texture, shape, and other morphological features in a random forest model largely following [23] and [24]. This study modifies these methods in a number of ways. Firstly, it employs kernel density estimation instead of histograms to account for samples in higher dimensions. Secondly, it is applied to segments that correspond to potato plants components instead of individual pixels. Lastly, it includes more class-specific indices from [13].

## 2. Materials and Methods

### 2.1. Study Site and Dataset

**Figure 1:**
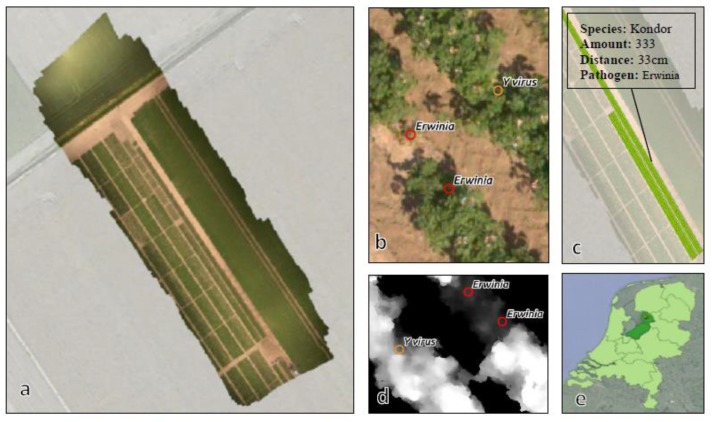
(**a**) RGB orthomosaic from the Hyperspectral Mapping System (HYMSY) under a Unmanned Aerial Vehicle (UAV) acquired on 19 June 2017; (**b**) RTK located diseased plants (430); (**c**) potato field information; (**d**) thresholded depiction of digital surface model (DSM) acquired on 19 June 2017; (**e**) location of the experimental field near Emmeloord in the Netherlands.

This research uses data that was originally made available for a joint experiment by Wageningen Plant Research (WPR) and the Laboratory of Geo-information Science and Remote Sensing of the Wageningen University, as part of the Ziekzoeker project (Figure 1). The dataset is comprised of VHR imagery which was captured and processed by the Hyperspectral Mapping System (HYMSY) [25] on 19 June 2017. This includes an 8.3 mm RGB orthomosaic, recorded by an onboard Panasonic GX1 camera with a 14 mm pancake lens (calibrated on-site with reference panels). The aerial images preceding the orthomosaic were stitched together through a process called structure-from-motion (Agisoft Metashape) which produced an 8.3 mm digital surface model (DSM).

The dataset covers roughly 0.5 hectare of an experimental potato field of the Dutch General Inspection Service (NAK) near Emmeloord (Netherlands). The field is level (<2 cm) which means that the DSM already contains absolute canopy height values. A multitude of potato species (*Solanum tuberosum*) were planted in this research area on May 11 including *Vermont*, *Kondor*, *Lady Claire*, and *Rosagold*. Several of these potato plants are known to be in various stages of infection with the Potato Y virus (specifically the *PVYNTN* strain) and the Erwinia bacteria. Field experts from NAK inspected these plants on a weekly basis throughout their lifespan and logged their locations with an RTK GNSS rover (0.02 m accuracy) if they exhibited symptoms of infection. Lab samples were taken to verify these observations, resulting in an exhaustive list (430) of diseased potato plants. Canopy patterns indicative of disease (e.g., necrosis and growth stunting) can also be seen in the VHR imagery, as the spatial resolution allowed for the distinction of objects roughly 10 cm in diameter (e.g., inflorescence; plants occlude each other).

### 2.2. Sampling and Modeling

Given the locations of diseased plants in the scene, healthy instances of potato plants were manually annotated in the VHR imagery. Only plants that were at least 2 full plants away from diseased instances (roughly 80 cm) were considered as candidates to ensure the same high degree of status certainty as the NAK. This mirrors the approach by [26] that used the same dataset to model potato disease.

From these locations, 170 healthy and 170 diseased potato plants were sampled on the criteria that they reflect the same variance of the full image (i.e., scene location, illumination, species, disease type, and severity). From the ‘diseased’ set, 10 points were randomly selected and buffered to 40 cm to incorporate all plant sizes. Within these buffers, manual segmentation was performed for the classes soil, flower, and veg. These segments are used as masks to extract the raster values from the VHR imagery (RGB + DSM) per class, on which 25 color transformations were performed (Table 1). The results are grouped into one dataset, referred to as *class data*, and normalized across all axes to fit in one byte (0–255) for easier parametrization.

**Figure 2:**
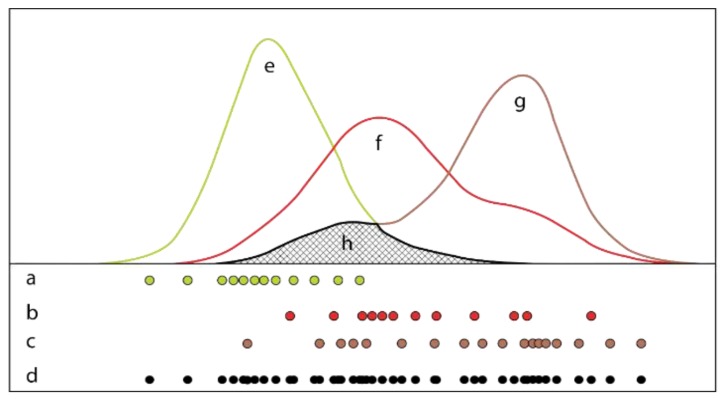
Approximation of three class distributions (**e**–**g**) with varying sample sizes (**a**–**d**) by kernel density estimation (KDE); the proposed method of evaluating separability between distribution sets, assessed as the ratio between distribution overlap (**h**) and their collective area (**e**–**g**) overlap in said distributions: (**a**) *veg* subset of *class data*; (**b**) *flower* subset of *class data*; (**c**) *soil* subset of *class data*; (**d**) collective *class data* in set dimension; (**e**) approximated *veg* distribution; (**f**) approximated *flower* distribution; (**g**) approximated *soil* distribution; (**h**) minimal overlap across all distributions.

Any object (or *class*) in any particular scene has an underlying probability distribution within the *n*-dimensional space defined by its color space [27]. The distributions for soil, flower, and veg were approximated by the sampling of their respective pixels (Figure 2). This allows for the definition of $p_{soil}\left(color\right)$, $p_{veg}\left(color\right)$, and $p_{flower}\left(color\right)$ as probability density functions (PDF) of color and height. Per Bayes rule, these function sets can be used as non-parametric Bayesian classifiers [22].

The samples in *class data* consists of 29 dimensions which allows for numerous definitions of *color* (Table 1). A limit of 5 dimensions per *color* definition was set to minimize information redundancy as they are highly correlated between each other. As *color* determines the appearance, and thus overlap, of all underlying distributions, its composition directly influences its viability as a classifier. Given *color*, kernel density estimation functions can approximate any distribution by:

(1) $${\hat{p}}_{class}\left(color\right)=\frac{1}{nh}\sum_{i=1}^{n}K\left(\frac{x-x_{i}}{h}\right),$$

where *h* denotes bandwidth size, *n* denotes the class-specific sample size, and *K* is a kernel function. As the former influences the estimate much more than the shape of the latter, Scott’s rule of thumb and a normal kernel are employed respectively [28,29]. The ability to adapt to complex shapes is important as those are common for most *color* transformations (Table 1).

**Figure 3:**
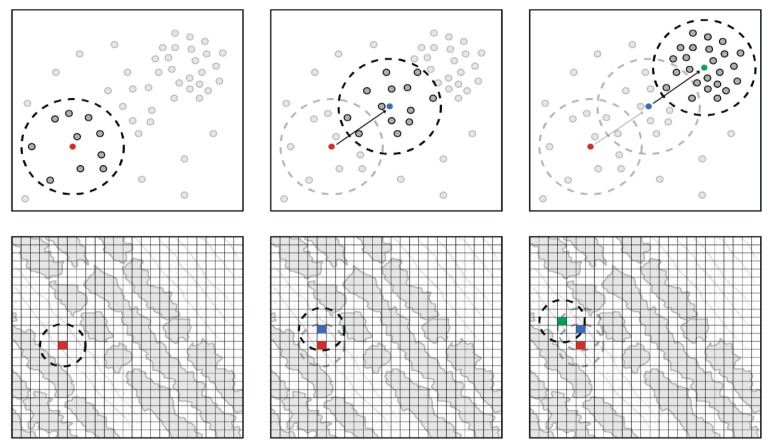
Visualization of the iterative process that underlies large scale mean-shift segmentation (LSMSS), where local modes for every pixel are found which ultimately segments the image. The top row depicts steps in feature space with their associative circles representing the spectral threshold, while the bottom row represent steps in image space with their associative circles representing the spatial threshold; red depicts any initial pixel from which other proximal pixels are selected (that adhere to both thresholds), a weighted mean (distance) is computed from this new set which points towards a new mode, this process is repeated until the mean and mode are approximately equal which indicates convergence; convergence ends with the spectral and spatial values of the found mode being assigned to the initial pixel (green and red respectively).

All 118,755 *color* definitions are evaluated on their ability to separate sampled class distributions, and on their performance as a model, for which two modified methods from [22] are employed. With $C=\left\{c_{1},\;..,\;c_{118755}\right\}$ denoting all *color* definitions, and $c=\left\{c^{soil},c^{flower},c^{veg}\right\}$ denoting class-specific samples, *separability* is measured per *c:*

(2) $$min=\int \min\left\{{\hat{p}}_{\left(c^{soil}\right)},\;{\hat{p}}_{\left(c^{flower}\right)},\;{\hat{p}}_{\left(c^{veg}\right)}\right\},$$

(3) $$\mathrm{total}=\mathrm{sum}\left\{\int {\hat{p}}_{\left(c^{\mathrm{soil}}\right)},\int {\hat{p}}_{\left(c^{\mathrm{flower}}\right)},\int {\hat{p}}_{\left(c^{\mathrm{veg}}\right)}\right\},$$

(4) $$overlap=\frac{min}{total},$$

where ∫(x) denotes integration by the trapezoid rule and *overlap* the ratio between overlapping distributions and their collective area. Model performance is evaluated by instancing training- and testing subsets per *color*, each following an 80:20 ratio. Confusion matrices per class are computed from these subsets and summarized by means of the Matthew correlation coefficient (MCC). This metric measures the quality of binary classification and was selected for its ability to account for varying class sample sizes [30]. The VHR imagery is clipped around the buffered locations (40 cm) of healthy (170) and diseased (170) potato plants, resulting in 340 raster images. These images are transformed to the *color* definition of the best performing model to separate the soil, flower, and veg instances within. They are also normalized to fit *class data* ranges, ensuring model applicability, and segmented by means of LSMSS (Figure 3).

**Figure 4:**
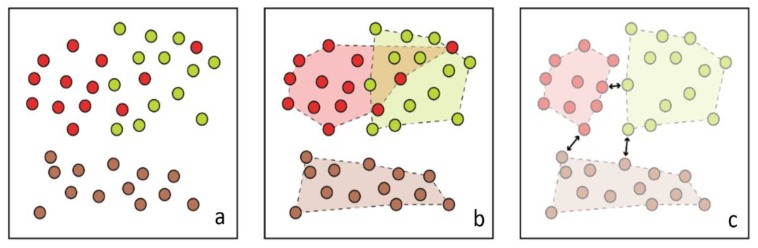
Visualization of spectral threshold estimation, depicted in 2D feature space (e.g., red and blue): (**a**) *soil*, *veg*, and *flower* data; (**b**) calculated convex hulls per class, used to determine and remove overlapping data from the smallest class; (**c**) estimation of minimal Euclidean distance between classes (i.e., selecting the shortest ‘arrow’) and setting it as *p*.

LSMSS requires spectral and spatial thresholds as set parameters that essentially define when pixels are to be considered similar. These parameters function as Euclidean thresholds that establish and iteratively expand segments as the associated pixels converge to their local modes (see [31] for a more in depth explanation). With $c=c_{s,\;n}$ denoting class-specific samples (*s*) in *n*-dimensional space, and $hull_{class}$ denoting a convex hull computed from $c^{class}$, the spectral threshold is estimated by:

(5) $$\mathbb{C}=\left\{c|c⊄hull_{s}\wedge c⊄hull_{f}\wedge c⊄hull_{v}\right\},$$

(6) $$p=\min\left(\begin{array}{c} \min\left\{d\left({\mathbb{C}}^{veg},\;{\mathbb{C}}^{soil}\right)\right\},\;\min\left\{d\left({\mathbb{C}}^{flower},{\mathbb{C}}^{soil}\right)\right\}\\ ,\;\min\left\{d\left({\mathbb{C}}^{flower},\;{\mathbb{C}}^{veg}\right)\right\} \end{array}\right),$$

where ℂ denotes *color* not found within the class-specific convex hulls, d denotes Euclidean distance function, and p the minimal Euclidean distance across all classes (visualized in Figure 4).

### 2.3. Initial Plant Mapping

The spatial threshold is fixed to the smallest class under consideration as the spectral and spatial ‘window’ in each iteration needs to contain a minimum of two classes as to not under-segment. This was determined to be *flower* as its class instances can generally be captured by 10 pixels (8.6 cm in diameter). Each segment in the resulting 340 segment sets, which contains the mean and variance of the underlying pixels, is classified as {*veg*, *soil*, *flower*} with the naïve Bayesian classifier previously associated with the selected *color* (Figure 2).

### 2.4. Class Expansion and Classification

Following overviews by [24] and [32], a list of 30 morphological features that were expected to capture plant deterioration was constructed (Appendix A). This was mainly done by means of a gray-level co-occurrence matrix (GLCM) from which *contrast* (*CON*), *dissimilarity* (*DIS*), *homogeneity* (*HOM*), *angular second moment* (*ASM*), *energy* (*ENG*), and *correlation* (*COR*) were computed. It was selected due to its frequent use in image analysis and is set to capture growth stunting and discoloration specifically [18,24,33].

Two GLCMs were computed from the DSM and hue (a spectral index used to isolate color perception, see [34]) in four directions (*0*, *45*, *90*, *135*) which are then summed to achieve directional invariance. This is done on two scales, 1 and 5 pixels, to capture their respective patterns, which results in 24 texture features. This list is further expanded with *volume*, *net area*, *#flowers*, *perimeter*, *aspect ratio*, and *solidity*, computed from the DSM and class objects respectively. The definitive list will consist of 170 *healthy* and 170 *diseased* features, each of them containing 30 morphological variables that are set to collectively approximate pathogen presence. It is then split into a training- and testing set to be used to feed a random forest model for disease detection.

This is largely following [23] who similarly employed texture analysis and random forest to classify semantic classes. A base model is trained using default parameters and used to iteratively optimize the hyperparameters of a new model, by randomly changing the initial values. Over-fitting and covariate redundancy in these models are accounted for by using N-fold cross validation on different compositions of the dataset. Performance of these models are quantified by computing their F1- and MCC scores:

(7) $$MCC=\frac{TP\times TN-FP\times FN}{\sqrt{\left(TP+FN\right)\left(TP+FP\right)\left(TN+FP\right)\left(TN+FN\right)}},$$

(8) $$F1=2/\left(\frac{1}{TP/\left(TP+FN\right)}+\frac{1}{TP/\left(TP+FP\right)}\right).$$

In these equations, TP represents true positives (what number of diseased plants are actually labelled as such), FP represents false positives (what number of diseased plants are incorrectly labelled as such), TN represents true negatives (what number of healthy plants are actually labelled as such), and FN represents false negatives (what number of healthy plants are incorrectly labeled as such). The variable importance of all features is also computed to quantify how much variance, which is expected to mainly come from disease status, is explained by what specific traits [35].

## 3. Results

### 3.1. Separability and Performance

**Figure 5:**
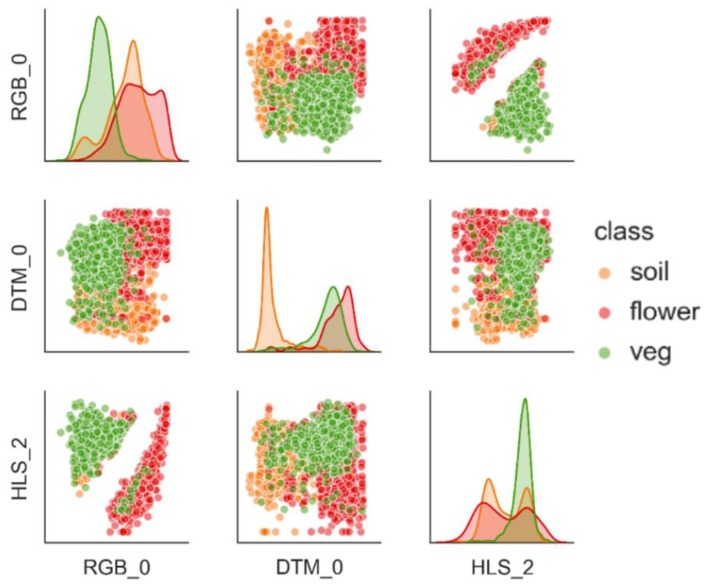
Scatter matrix of 3 dimensions (out of 30) with class data, where the diagonal axis depicts the underlying class distributions through kernel density estimation (KDE); notice the varying shapes and overlap per *color*, and how they emphasize different spectral characteristics.

The sampled *class data* totaled 43,287 vectors of which 68.1% are labeled veg, 29.6% are soil, and 2.3% are flower. This imbalance does reflect the scene composition but could hamper classification if class distributions are not distinctive enough. Figure 5 suggests that this is not the case as it depicts varying separability that varies considerably per *color* definition. The plot involving $HLS_{2},\;RGB_{0}$ (i.e., red intensity) for instance, clearly separates *flower* and *veg* but with some outliers suggesting faulty or unbalanced sampling (as they introduce *false* overlap). The cluster shapes roughly follow the preceding (linear and non-linear) transformations, but also show considerable correlation between some dimensions, as they are ultimately only computed from 3 bands that come from the same distribution.

The separability per *color* definition was quantified, as was their performance as naïve Bayesian classifiers (Table 2). It shows that particular dimensions in isolation do not allow for adequate class distinction, as the associated spectral characteristic need not be significant in all classes, but can add to an overall distinction if combined with other (more expressive or more general) dimensions (e.g., $LUV_{1}$ as seen in Table 2). Overlap between distribution sets are also considerably lower in higher dimensions (limited to sets of 5 as per their expected correlation). As the terrain is level, hue and height ($DSM_{0},\;HSV_{0}$) in isolation intuitively do offer the best separation as vegetation is physically higher and classes exhibit varying dominant colors.

This also persists in their performance as a model, where soil is the best classified by height, flower is best classified by ‘blue-ness’, and veg is best classified by ‘green-ness’. Slightly different compositions do surface here, confirming that separability indeed does not necessarily equal good classification [27]. It also shows that *flower* exhibits lower MCC results across all models, again indicating either faulty sampling or broad class definitions. *Color* composed of height, saturation, hue, lower wavelengths, and luminance, best captures *veg*, *flower*, and *soil*.

**Figure 6:**
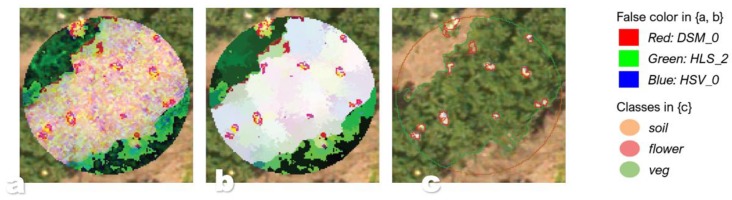
One example of the 340 approximated plant objects following LSMSS: (**a**) *color* raster clip depicted in false color; (**b**) LSMMSS-segmented *color* clip depicted in false color; (**c**) vector object containing *veg* (*green*), *soil* (*brown*), and *flower* (*red*) after *color* clip classification; *veg* and *soil* does show accurate segmentation with occasional misclassification of *flower* as suggested by Table 2; note that the spectral threshold appears valid as there is now less within-class variance in all classes without removing discrete boundaries.

All sampled plant locations were then used to clip (1 m in diameter) 340 discrete raster images that each contain one plant instance. All images were then transformed to the most separable *color*, segmented, and classified using the model associated with said *color* (Figure 6).

The same performance ratio can be expected as the samples were varied and the imagery was homogenous, but with lower results overall simply due to the unit size (i.e., bundled pixels would inherently influence any metric more than individual pixels). The spectral and spatial thresholds appear to be valid as all classes appear much more homogenic (minimized within-class variance) without losing their minimal spectral boundaries (Figure 6).

### 3.2. Disease Classification

Given these 340 object-based approximations of potato plants, 30 morphological features were computed for each. Initial evaluation of this data showed different means for all features, of which 24 were significant as determined with T-tests (Appendix A). This data was split into a training and testing set (0.8–0.2), of which the former was used to initialize a random forest model with default parameters for disease status (Table 3).

Although this base model was only instanced to evaluate hyperparameters and/or feature composition to be adjusted in subsequent models, the initial results already suggest that disease classification using only morphological traits is possible with F1 and MCC values averaging around 0.70 and 0.41, respectively.

**Figure 7:**
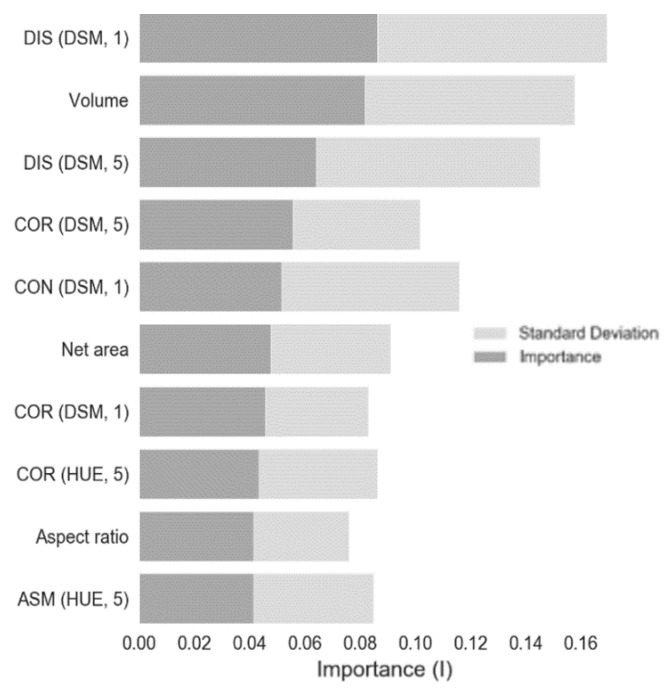
Variable importance subset: only the 10 highest instances of 30 morphologic traits are shown (see [35] for a more in-depth explanation of variable importance).

Following the optimization process and *n*-fold cross validation, the optimal hyperparameters only differed in tree amount (10 to 100) and maximum feature size (all to 20), but improved results by a factor of 0.2. The fact that considerably more trees are employed suggests that either the feature set exhibits minute differences important to disease classification that cannot be captured by low tree amounts, or simply that the two feature datasets (healthy and diseased) overlap, resulting in arbitrary leaf splits giving a false sense of information gain. The latter is supported by the low individual feature importance values (Figure 7), and an earlier statement about the sheer subtlety of disease traits. The fact that the most expressive traits are derived from $DSM_{0}$ therefore might not equate to much given their minute differences. The model itself performs well, which is indicative of deterministic properties in the collective feature set.

## 4. Discussion

The results presented show that the approach proposed was able to classify potato plant pathogens through morphologic features. Manual segmentation was carefully performed (e.g., shifting local contrasts to better approximate class boundaries), but ‘clean’ supervised samples that are completely separable in appropriate *color* definitions could not be guaranteed (Table 2). Although this is arguably reflecting real use cases that could employ flawed sampling schemes, the decision to use a naïve classifier in combination with faulty sampling could result in ambiguous class definitions that hinder classification. The normalization of *class data* across all axis could have also resulted in loss of detail, as some non-linear transformations resulted in kurtosis. It should however also have accounted for any sensitivity to luminance, surface orientation, and other photographic conditions that may occur across the samples (Table 1). This sensitivity is an important criticism to the original RGB space, but one that is accounted for by the normalized *rgb* color model [27,36,37] which makes the RF model more robust.

In comparison to more conventional segmentation methods that employ fixed spectral indices (e.g., Otsu thresholding with ExG), this approach is more class-invariant as it effectively tries to find the *n*-dimensional *color* combination that best separates the objects of interest. This is increasingly relevant as spatial resolution increases, which only exacerbates the pixel variability difficulties echoed by [5,13].

Fixed (or even adaptive) thresholding do not allow for these minute local differences, LSMSS does however as it bundles pixels on local similarities. It was not possible to fully prevent class overlap which either means that class definitions are too broad (and thus, exhibit *genuine* overlap) or that the sampling scheme is flawed (which introduces *false* overlap). The pixel sets related to this overlap (i.e., equal probability of pixels belonging to two or more classes) were removed, as illustrated in Figure 4, because if no ‘safe’ spectral threshold can be determined, LSMSS would only establish individual pixels as segments, effectively devolving it to a pixel-based classifier. It was decided to remove the pixel sets from the smallest class in favor of computation speedup (less data to evaluate), which discards already scarce class information. This paper subscribes to the theorem of [38] which states that complex (or subtle) classes require more semantic knowledge, which makes sampling costly and exhaustive sample sizes rare, ultimately resulting in imbalanced sample sizes. This was one of the reasons why kernel density estimation (KDE) was employed for *color* evaluation, instead of histograms like [22], because KDE essentially interpolates (and extrapolates) between given samples, always giving probability estimates.

The model performs well despite low individual importance (Table 3; Figure 7). This means that the model does capture plant pathogens, confirming the quality of the disease evaluation by NAK, but also that the individual features vary considerably not only due to the sheer subtlety of the traits per pathogen and the unknown extent of infection, but also due to the varying properties of the given potato plants themselves. The potato plants depicted in the dataset have grown to physically overlap making for ambiguous class boundaries. Due to the principles of good continuation and good form respected by the human visual system (gestalt principles, see [17]), human interpreters can imagine their boundaries as the plants exhibit recurring patterns (e.g., volume, center of mass). This paper arguably circumvented this problem as locations of (diseased) plants were known, as required for supervised classification, but similarly detailed location data are not commonly available. This is also seen in [39] where an arbitrary window is enforced in which object-based thresholding is performed to detect potato pathogens. This particular study does not incorporate object-specific traits (shape, texture, color) making it unfit for our problem specification. Moreover, common techniques for vegetation detection like the work presented by [40] are limited to a dual-class problem and cannot be applied to more semantic classes.

Our approach fully explores the VHR UAV imagery and provides accuracy (Table 3) values that fall within the accuracy range of other disease detection methods found in literature [5]. However, as was also indicated by [7], the application of VHR UAV imagery for disease detection is still in the research phase and has not reached its full potential yet. Future research should be on the fusion of VHR RGB imagery with hyperspectral and/or thermal imagery in combination with advanced data analysis methods to distinguish between diseases and improve the early detection potential [41].

## 5. Conclusions

Summarizing, this paper has shown how color transformations can improve object segmentation, and that morphological traits computed from said objects could be used to find proxies for subtle biophysical processes such as disease. This is however a matter of semantics, or a user-driven set of conditions, and ultimately requires more advanced class modeling to more reliably isolate the more complex classes (i.e., not only exploiting spectral information). Per the theorem of [38], this can only be done with more accurate and reliable data (e.g., disease severity).

Precautions have been taken to generalize the model (e.g., color normalization and varied samples) and there are other relevant studies that have also built models on single observation days [19,42]. However, more data is also recommended to further validate the robustness of the model.

Additional research is recommended in the field of fuzzy classification, moment-based shape descriptors, hyperspectral indices, to enable more advanced object-based class modeling. [18,43,44] give further suggestions on how invariant object recognition and fuzzy classifiers could mimic the human visual system for advanced class modeling.

## Figures and Tables

**Table 1:** Color transformations and their purpose.

Transformation	Purpose	#	Source
**rgb**	Normalize luminance	3	[27]
**YUV**	Compression (video)	3	[27]
**HSV**	Intuitive color representation	3	[27]
**HLS**	Intuitive color representation	3	[27]
**LAB**	Uniform color representation	3	[27]
**LUV**	Uniform color representation	3	[27]
**XYZ**	Modeling the human retina	3	[27]
**I1I2I3**	Decorrelate primary colors	3	[27]
**EXR**	Emphasize red tones	1	[13]
**EXB**	Emphasize blue tones	1	[13]
**EXG**	Emphasize green tones	1	[13]
**CIVE**	Emphasize green tones	1	[13]

**Table 2:** Separability and model performance per *color* definition (notated as ‘dimension_band’ such as DSM_0) in sets of 5, 3, and 1; note how *flower* exhibits lower classification results overall.

*#*	*Color Definition*	*MCC*(*veg*)	*MCC*(*Flower*)	*MCC*(*Soil*)	*MCC*(*µ*)	*Overlap*
*5*	DSM_0,HLS_2,HSV_0,LUV_1,LUV_2	0.860262	0.649457	0.883537	0.51032	0.002226
	DSM_0,YUV_2,HLS_2,HSV_0,LUV_2	0.857509	0.63696	0.881851	0.50556	0.002472
	DSM_0,I23_1,I23_2,LUV_1,LUV_2	0.845499	0.625602	0.869054	0.49758	0.001609
	DSM_0,YUV_2,HLS_2,HSV_0,YUV_1	0.854386	0.604864	0.881494	0.49469	0.002744
*3*	DSM_0,HSV_0,HLS_1	0.834355	0.537583	0.871446	0.46829	0.00611
	DSM_0,YUV_0,HSV_0	0.835072	0.535683	0.868721	0.46711	0.005786
	RGB_1,DSM_0,HSV_2	0.838721	0.523977	0.873642	0.46484	0.003734
	RGB_0,DSM_0,HSV_2	0.775362	0.55155	0.814206	0.45313	0.003568
*1*	DSM_0	0.6276	0.19633	0.83016	0.32548	0.021265
	RGB_2	0.57866	0.43062	0.50568	0.32044	0.100627
	XYZ_2	0.57112	0.42312	0.49702	0.31516	0.100731
	HSV_0	0.70743	0.13215	0.79449	0.30469	0.047911

**Table 3:** Random forest model parameters and performance (evaluated with F1 and Matthews Correlation Coefficient (MCC) that range from 0 to 1, Gini is used to quantify information gain); The optimized model exhibits slightly higher values but with considerably more trees, which also implies some feature redundancy.

		F1	MCC	Model Parameters
base model	healthy	0.73		trees = 10 bootstrap = true max features = none minimal split size = 2 minimal leaf size = 1
	diseased	0.68		
	avg/total	0.7	0.41	
optimized model	healthy	0.75		trees = 100 bootstrap = true max features = 20 minimal split size = 2 minimal leaf size = 1
	diseased	0.72		
	avg/total	0.73	0.47	

## Appendix A

**Table A1:** Mean feature list for diseased and healthy objects: *T* tests are performed on every feature and displayed with their associated *P* values (indicating significance). Significant *T* values are highlighted with a bold font (*P* ≤ 0.05).

	Healthy (µ)	Diseased (µ)	*T* Values	*P* Values
*Volume*	72.7668	61.566	**7.44281**	0
*# Flowers*	7.64118	6.38235	2.78653	0.00564
*Aspect Ratio*	1.06597	1.06297	0.39755	0.69125
*Solidity*	0.05259	0.06375	**−2.217**	0.02733
*Net Area (m^2^)*	0.50749	0.46247	**6.11125**	0
*Perimeter (m)*	6.22416	5.7683	**3.29756**	0.00108
*Contrast 1 (DSM)*	111.887	140.36	**−6.0246**	0
*Dissimilarity 1 (DSM)*	5.00174	5.56444	**−5.9483**	0
*Homogeneity 1 (DSM)*	0.33563	0.32129	**4.3921**	0.00002
*ASM 1 (DSM)*	0.001	0.00082	**5.26375**	0
*Energy 1 (DSM)*	0.03113	0.02835	**5.40393**	0
*Correlation 1 (DSM)*	0.9672	0.96978	−2.6944	0.0074
*Contrast 5 (DSM)*	759.653	933.711	**−5.9451**	0
*Dissimilarity 5 (DSM)*	17.7592	19.8636	**−6.1313**	0
*Homogeneity 5 (DSM)*	0.07983	0.07286	**4.44554**	0.00001
*ASM 5 (DSM)*	0.00033	0.00029	**4.0238**	0.00007
*Energy 5 (DSM)*	0.01786	0.01686	**4.04216**	0.00007
*Correlation 5 (DSM)*	0.75588	0.78134	**−4.2657**	0.00003
*Contrast 1 (Hue)*	30.6429	31.2285	−0.5783	0.56345
*Dissimilarity 1 (Hue)*	3.92445	3.97107	−0.7314	0.46502
*Homogeneity 1 (Hue)*	0.24755	0.24455	0.84087	0.40102
*ASM 1 (Hue)*	0.00406	0.00368	**3.21612**	0.00143
*Energy 1 (Hue)*	0.06311	0.06009	**3.37492**	0.00082
*Correlation 1 (Hue)*	0.64758	0.68589	**−4.8532**	0
*Contrast 5 (Hue)*	67.2367	71.6527	**−2.4429**	0.01508
*Dissimilarity 5 (Hue)*	6.08534	6.30297	**−2.7502**	0.00628
*Homogeneity 5 (Hue)*	0.16461	0.15893	**2.96378**	0.00326
*ASM 5 (Hue)*	0.00313	0.00277	**4.29112**	0.00002
*Energy 5 (Hue)*	0.05551	0.05213	**4.48815**	0.00001
*Correlation 5 (Hue)*	0.19399	0.2515	**−5.3375**	0
